# Editorial

**Published:** 2015-01-06

**Authors:** Yoichi Hiasa

**Affiliations:** Professor, Department of Gastroenterology and Metabology Ehime University Graduate School of Medicine Shitsukawa, Toon, Ehime 791-0295, Japan

Dear Colleagues,

In this evolving world, when information is piling up from different corners, scientific journal represents one of the most important avenues of transmission of knowledge from the sources to the users. In fact, the need of scientific journals in developing countries is enormous, but very few are coming out from there. This is more relevant in the field of gastroenterology and hepatology. In one hand, the numbers of patients with gastroenterological and hepatological problems are on rising trend in these parts of world. On the contrary, many of these patients are not able to follow the treatment guideline provided by advanced and developed countries. These facts indicate that there should be ongoing investigations about epidemiology, pathogenesis, prevention and therapy of different diseases within the disciplines of gastroenterology and hepatology to optimize with the need of these patients at developing countries. The retrieved information should reach the hands of physicians policy makers, and medical associates of these regions.

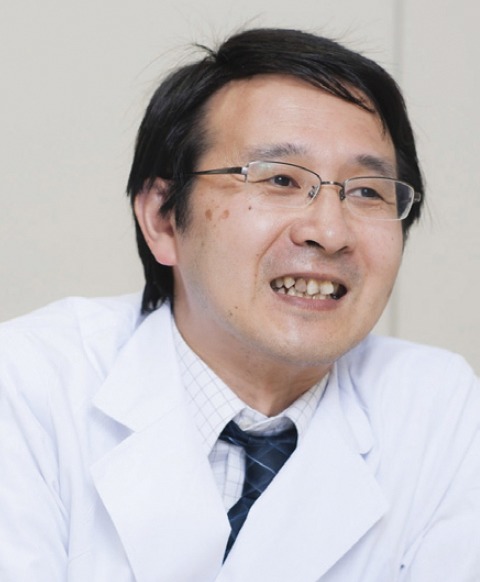



To my understanding, the ‘Euroasian Journal of Hepato-Gastroenterology’, that is endorsed by liver organizations of more than 10 countries, deserves a specific place among the journals of these disciplines. In most cases, a journal of a discipline is endorsed by usually one organization. The Euroasian Journal of Hepato-Gastroenterology is going to complete its 4 years of regular publication, a significant event for any journal of this region. Also, conferences and seminars of this area are well represented in this journal.

Standing at the footstep of 5th year of its publication, I extend my congratulation to the contributors, editors and publishers of the journal. I am sure that the journal would soon be reflected as a prominent journal of this region.

**Yoichi Hiasa, **MD PhD

Professor

Department of Gastroenterology and Metabology

Ehime University Graduate School of Medicine

Shitsukawa, Toon, Ehime 791-0295, Japan

